# The Expression of Small Regulatory RNAs in Clinical Samples Reflects the Different Life Styles of *Staphylococcus aureus* in Colonization vs. Infection

**DOI:** 10.1371/journal.pone.0037294

**Published:** 2012-05-22

**Authors:** Juan Song, Claire Lays, François Vandenesch, Yvonne Benito, Michèle Bes, Yonglie Chu, Gérard Lina, Pascale Romby, Thomas Geissmann, Sandrine Boisset

**Affiliations:** 1 Department of Immunology and Pathogen Biology, Medical school of Xi'an Jiaotong University, Xi'an, Shaanxi province, China; 2 Université de Lyon, Université Lyon1, Lyon, France; 3 INSERM U851, Lyon, France; 4 Hospices Civils de Lyon, Centre National de Référence des Staphylocoques, Bron, France; 5 Université de Strasbourg, CNRS UPR 9002, IBMC, Strasbourg, France; University of Liverpool, United Kingdom

## Abstract

Small RNAs (sRNAs) are involved in the post-transcriptional regulation of metabolic pathways and in responses to stress and virulence. We analyzed the expression levels of five sRNAs of *Staphylococcus aureus* during human colonization or infection. Total RNA was isolated from nasal carriers, abscesses and cystic fibrosis patients (20 subjects per condition). The expression levels of the sRNAs were measured in the clinical samples and compared with those of the corresponding strains grown *in vitro*. Five sRNAs were encoded and expressed in all clinical strains *in vitro*. *In vivo*, the global expression of the five sRNAs was extremely variable in the abscessed patients, more homogeneous in the cystic fibrosis patients, and highly uniform in the nasal carrier samples. The expression levels of the sRNAs *in vivo* resembled those obtained at exponential phase or late exponential phase of growth *in vitro*, for three and one sRNA respectively; while for one sRNA, the expression was always higher *in vivo* as compared to *in vitro* growth. The *in vitro* conditions do not uniformly mimic the *in vivo* conditions for sRNA expression. Nasal colonization is associated with a unique expression pattern of sRNA that might reflect the commensalism of *S. aureus* in this niche.

## Introduction

*Staphylococcus aureus* is both a major human pathogen and a ubiquitous commensal belonging to the normal human flora. Approximately 20% of the healthy population is colonized asymptomatically with *S. aureus* in the nostrils without any associated disease [1]. However, this pathogen is also responsible for a wide range of human diseases, from minor skin infections to life-threatening diseases, such as endocarditis, pneumonia, and septic shock. It is also one of the leading causes of hospital-acquired infections, often causing post-surgical wound infections. The wide diversity of clinical infections caused by *S. aureus* depends on the expression of numerous virulence factors, including toxins, adhesins, enzymes, and immunomodulators [2]. These proteins are expressed in a coordinated manner under the control of multiple regulators, such as two-component systems, transcriptional regulatory proteins, secondary metabolites, small peptides and regulatory RNAs [3], [4].

The role of small RNA (sRNAs) in gene regulation has been established in bacteria [5]–[8]. In *S. aureus*, RNAIII was the first regulatory RNA reported; it has the unique property of acting both as a mRNA that encodes the 26 aa delta hemolysin (PSM) peptide, and as the effector molecule of the accessory gene regulator (*agr*) system that represses early virulence factors and activates post-exponentially expressed exotoxins [9], [10]. Hence, RNAIII plays a key role in the quorum sensing-dependant regulatory networks and coordinately regulates the synthesis of virulence-associated genes and the transcriptional regulator Rot at the post-transcriptional level [11]–[14]. In recent years, bioinformatic approaches coupled with expression analysis [15]–[17], cloning/sequencing of small cDNAs [18] and high throughput sequencing [19], [20] have revealed several classes of regulatory RNAs, including sRNAs, *cis*-encoded antisense RNAs and *cis*-acting regulatory regions of mRNAs. Most of these RNAs are encoded by the core genome, while several sRNAs are expressed from mobile elements, pathogenic islands [16], [20] and plasmids [21].

The expression of several sRNAs has been monitored *in vitro* using different methods, such as Northern blot or RT-PCR. So far, no studies have investigated the expression of these sRNAs in animal models or human infections, except for RNAIII [22], [23]. Using real-time reverse transcriptase PCR (RT-PCR), the yield of RNAIII was measured in the sputum of cystic fibrosis (CF) patients colonized with *S. aureus*. These data revealed that RNAIII was poorly expressed in the sputum, and no correlation existed between *S. aureus* density and RNAIII expression [22]. Furthermore, Loughman *et al.* [23] showed that the RNAIII transcript level was reduced in a human cutaneous abscess as compared with the levels obtained for the corresponding strain at the stationary phase of growth. However, in invasive abscesses, RNAIII was detected at a level comparable to that of the corresponding strain at the stationary phase [23]. In the case of nasal colonization, few data document RNAIII expression. Burian *et al.* [24] performed an analysis of four nasal carriers and showed that *agr* was not activated during nose colonization. Taken together, these data show that it is difficult to predict the expression of sRNAs *in vivo*. It is likely that depending on the clinical context, the host-pathogen interactions influence bacterial gene expression.

In the present study, we determined the expression of several *S. aureus* sRNAs during human colonization and infection. To this end, three different clinical specimens were chosen, namely nose swabs, sputum from cystic fibrosis patients, and pus from an acute suppurative cutaneous infection (i.e., abscess). We used RT-PCR to quantify the well-characterized RNAIII and four recently described sRNAs (RsaA, RsaE, RsaG and RsaH), representing the diversity of expression *in vitro* [15]. These four sRNAs represented a class of stable and structured non coding RNAs which are expected to regulate gene expression by a shared mechanism, e.g. mRNA binding as it was shown for RNAIII [15].

## Results

### Characteristics of the clinical isolates cultured from the 60 patients

**Figure 1:**
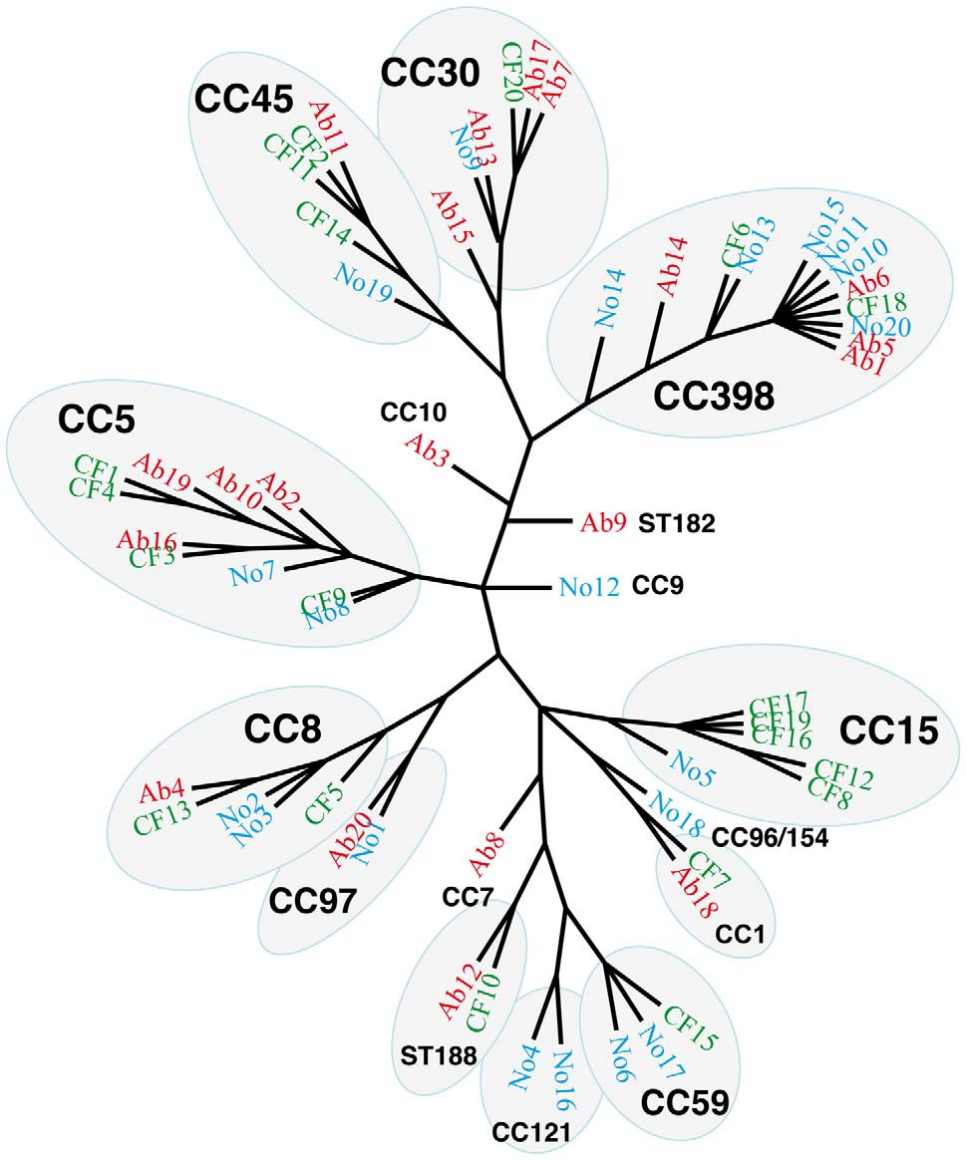
Split network tree constructed from the results of the microarray hybridization analysis of the 60 isolates. The tree was constructed using raw data from the microarrays to visualize the distribution of the strains among different Clonal Complexes. The strains were assigned the following numbers: Ab1 to Ab20 (in red), abscess strains; CF1 to CF20 (in green), cystic fibrosis sputa strains; No1 to No20 (in blue), nasal isolates. Multilocus sequence types (ST) and/or clonal complexes (CC) are indicated.

The 60 specimens represented the three categories of *S. aureus*-human interaction, namely nasal colonization (20 cases), chronic infection in cystic fibrosis patients (20 cases) and acute suppurative cutaneous infection (20 cases). The vast majority of the isolates were methicillin susceptible (57/60; Table 1), which is consistent with the low prevalence of community-acquired, methicillin-resistant *S. aureus* in France [25]. All 60 isolates were genotyped using DNA microarrays, targeting 180 genes and 300 alleles (Alere Technologies). Genotyping was a prerequisite to rule out any differences in the *in vitro* expression profiles of the sRNAs that could reflect differences in the genetic background of the corresponding isolates. The results showed that the 60 isolates belong to various clonal complexes (CCs), and the observed CCs were equally distributed among the three groups (Table 1). An in-depth analysis using the combinatorial data obtained from the 180 genes showed that each branch of the tree obtained from the 60 isolates most often contained isolates from the three groups of infection (Figure 1).

**Table 1:** Characteristics of *Staphylococcus aureus* isolates.

*Clonal complex/Agr type*	*Methicillin susceptibility (Number of isolates)*	*Samples types*		
		Abscesses*	CF sputa*	Nose swabs*
**CC1/III**	S (2)	1	1	
**CC5/II**	S (9) R (1)	4	4	2
**CC7/I**	S (1)	1		
**CC8/I**	S (3) R (2)	1	2	2
**CC9/II**	S (1)			1
**CC10/II**	S (1)	1		
**CC15/II**	S (6)		5	1
**CC30/III**	S (6)	4	1	1
**CC45/I**	S (5)	1	3	1
**CC59/I**	S (3)		1	2
**CC96/154/III**	S (1)			1
**CC97/I**	S (2)	1		1
**CC121/IV**	S (2)			2
**CC398/I**	S (12)	4	2	6
**ST182/I**	S (1)	1		
**ST188/I**	S (2)	1	1	
		20	20	20

* n = number of isolates.

### The expression of the four sRNAs is different during culture

**Figure 2:**
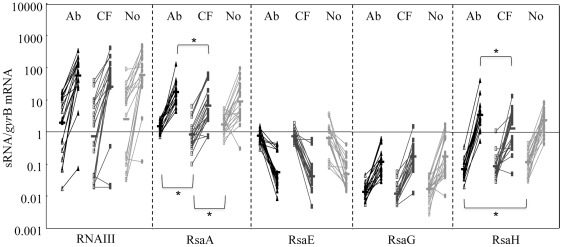
Kinetics of sRNA expression *in vitro*. Clinical *S. aureus* strains were grown in BH media until mid (OD_550_ = 0.5) (open triangle) or late-exponential (OD_550_ = 6) (plain shape) growth phases. Total RNA was extracted, and the sRNA transcripts were quantified by RT-PCR normalized to the level of *gyr*B mRNA expression. The results are presented according to the isolation sites of the 60 strains: 20 abscesses (Ab), 20 cystic fibrosis sputa (CF) and 20 nasal (No). The results represent the mean of 2 replicate experiments. The bars indicate geometric mean values. *For each sRNA, statistically significant differences (Mann Whitney, p<0.05) between the level of expression in mid or in late-exponential growth phase and the origin of the isolate are indicated.

The expression of sRNAs in the 60 clinical isolates was assessed using RT-PCR at the mid (OD_600_ = 0.5) and late exponential phase (OD_600_ = 6) of growth in rich medium (BH). Notably, the growth curves of the 60 isolates were extremely similar (not shown). The expression level of each sRNA was normalized against *gyr*B mRNA, which is constitutively expressed, and thus was used as a calibrator [23]. The experiments showed that each sRNA has a different expression profile. RNAIII and RsaA were highly expressed, while RsaE and RsaG were both poorly expressed. As shown in Figure 2, RNAIII, RsaA, RsaG and RsaH accumulated in the late-exponential phase, as observed for the laboratory strains RN6390 (*sig*B deficient) and HG001 (*sig*B repaired) (Figure S1). Conversely, the yield of RsaE was significantly reduced post-exponentially in the clinical isolates but not in the laboratory strains (Figure S1). The average level of expression for RNAIII, RsaE and RsaG was similar regardless of the *S. aureus* isolate origin (cutaneous abscesses, cystic fibrosis sputa or nasal swabs), whereas the average level of expression for RsaA and RsaH was slightly different among the *S. aureus* strains. For example, in mid-exponential phase, the average level of RsaA was statistically lower in the isolates from the cystic fibrosis sputa than in those from the abscesses (p = 0.013) and nose (p = 0.016). The expression of RsaH was statistically lower in the abscess isolates in the mid-exponential phase as compared with the nasal isolates (p = 0.030). In addition, the expression of RsaH was higher in abscess isolates in the late-exponential phase compared with the CF isolates (p = 0.037).

### The patterns of sRNA expression during *in vitro* culture reveal relatively high and low sRNA-producing isolates

**Figure 3:**
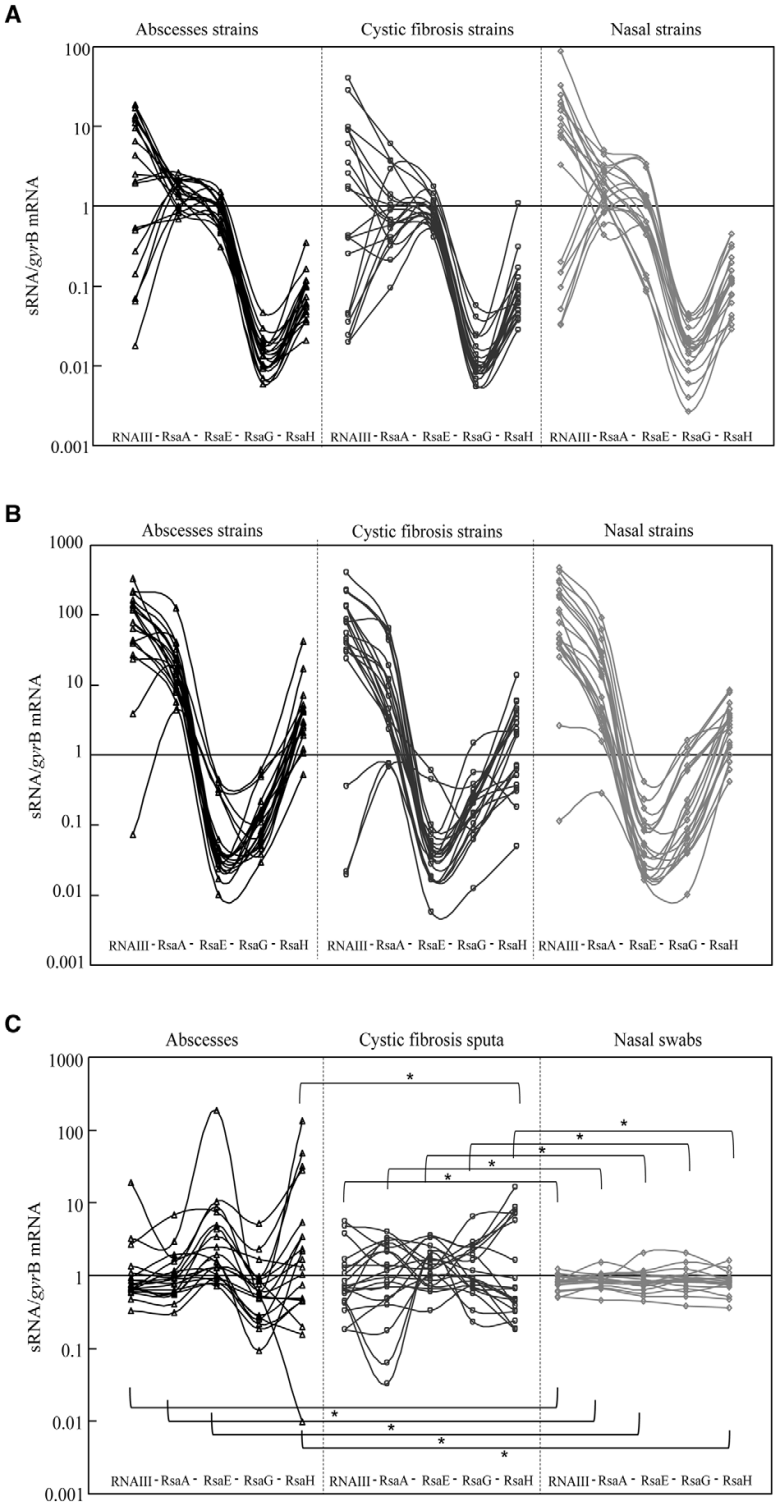
Expression of *S. aureus* sRNAs *in vitro* and *in vivo*. The expression of the sRNAs was quantified and normalized to the level of *gyr*B mRNA expression after *in vitro* culture to the mid- (panel A) or late-exponential phase (panel B) for the 60 strains, as described in Figure 2, and directly in the 60 clinical samples (panel C). The values from two separate RT-PCR experiments were used to calculate the mean expression for each point. *For each sRNA, statistically significant differences (Levene, p<0.05) between the distribution of the expression levels and the origin of the isolate are indicated.

For a given isolate, there was a correlation of the sRNA expression levels between the mid and late-exponential phases; the isolate exhibiting a low expression level at the mid-exponential phase also exhibited a low level of expression at the late-exponential phase (Figure 2). When considering the distribution of expression levels for the five sRNAs, each sRNA could be characterized according to the diversity of the expression level that remained consistent regardless of the clinical origin of the isolates (Figures 3A and 3B). The expression level diversity appeared to be narrow for RsaE, RsaG and RsaH and slightly wider for RsaA, especially in the CF and nasal isolates. In contrast, the distribution of RNAIII expression was wide in all clinical situations during the mid-exponential phase, but the expression profiles were similar among the isolates grown during the late-exponential phase. Interestingly, for a given isolate, we observed a link between the expression levels of the different sRNAs regardless of the origin of the isolates, e.g., a low RNAIII-expressing isolate also appeared to express low levels of RsaA, E, G and H (Figures 3A and 3B). Because these results were established relative to *gyr*B mRNA, we asked whether these differences reflected a true variation in sRNA expression levels or whether the differences were due to variations in *gyr*B mRNA. To this end, two additional housekeeping sRNA genes were selected (4.5S RNA and RNase P) and used as calibrators to assess the level of expression of RNAIII in 9 isolates chosen as representatives of the expression level diversity observed with calibrator *gyr*B. This experiment revealed that regardless of the calibrator used, each isolate displayed a similar pattern of expression, i.e., the low RNAIII expression observed using *gyr*B as a calibrator was also observed when 4.5S and RNase P were used as calibrators (Figure S2).

### The expression of sRNAs in clinical samples

To monitor sRNA expression in the host, we analyzed direct transcripts from clinical samples using RT-PCR. The results were normalized against the *gyr*B gene, as in the *in vitro* studies. All five sRNAs could be detected in the clinical samples, thus confirming their *in vivo* relevance. Strikingly, the expression profiles observed *in vivo* (Figure 3C) differed from those *in vitro* (Figures 3A and 3B). Furthermore, in contrast with the results obtained *in vitro*, there was no link between the expression levels of the various sRNAs among the isolates *in vivo* (Figure 3C). The expression levels for the five sRNAs were extremely variable in abscess samples, more homogeneous in the cystic fibrosis samples, and uniform in the nasal samples (Figure 3C). When comparing the abscess and CF sputa samples, there were no significant differences in the levels of sRNA expression, except for RsaH (p<0.01). When comparing abscess and nasal samples, the magnitude of the expression was significantly different for RNAIII, RsaA, RsaE, and RsaH (p<0.05). A comparison of the CF sputa and nasal samples revealed that the magnitude of the expression was significantly different for all five sRNAs (p<0.001).

### Comparison of the sRNA expression in bacterial cultures and human samples

**Figure 4:**
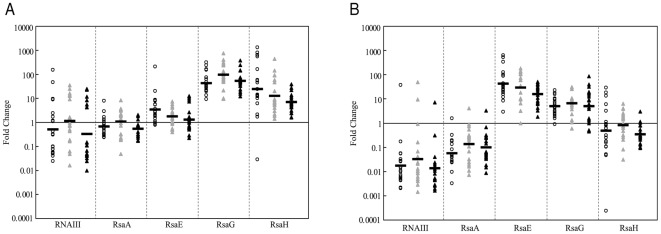
Comparison of sRNA expression in bacterial cultures and human samples. The results are expressed as the ratio of mRNA expression levels *in vivo versus* those *in vitro*. (**A**) Fold change in transcript levels for each sRNA in the human samples (circle: abscesses; grey triangle: cystic fibrosis sputa, black triangle: nasal) relative to the levels in bacteria at the mid-exponential growth phase in BH media. (**B**) Fold change in transcript levels for each sRNA in human samples relative to the levels in bacteria at the late-exponential phase in BH media. The bars indicate the geometric mean values.

We compared the sRNA expression levels in each sample with those in the corresponding *S. aureus* strain grown to mid and late-exponential phases *in vitro* (Figures 4A and 4B). These results show that the yields of RNAIII, RsaA and RsaE in the human samples resembled those obtained *in vitro* during mid-exponential phase of growth. The level of RsaH expression in the clinical samples corresponded better with that obtained *in vitro* during the late-exponential phase of growth. However, for RsaG, the expression observed in the infected samples was higher than that obtained at all phases of *in vitro* growth (Figures 4A and 4B).

## Discussion

In general, sRNAs are involved in the post-transcriptional regulation of different cellular pathways, and some of them have been implicated in virulence (for a review, see [26], [27], [28]). Most *S. aureus* sRNAs have been identified from a limited number of laboratory strains, and their expression profiles in human remain unknown, except for RNAIII, for which the expression in clinical samples, such as cystic fibrosis sputa or nasal secretions, has been studied [22]–[24], [29]. The aim of this study was to determine whether sRNAs are present in clinical isolates, and if their expression is dependent on colonization or infection in humans. To this end, we focused on four recently described sRNAs, representing a diversity of expression patterns [15], and the well-characterized RNAIII from *S. aureus*. Two clinical specimens, representing a diversity of clinical conditions were chosen, namely sputum from cystic fibrosis patients (chronic infection, 20 cases) and pus from acute suppurative infections requiring surgical drainage (20 cases). We also analyzed 20 cases of *S. aureus* nasal colonization. We observed that all *S. aureus* isolates were expressed in all five sRNAs after culture in BH medium to mid or late-exponential phases of growth. This observation was previously well documented for RNAIII, which is encoded by virtually all *S. aureus* isolates [30]. The observation that all the clinical isolates encoded the other four sRNAs (RsaA, RsaG, RsaE, RsaH), which were expressed at various levels, suggests that these sRNAs are expressed in the core genome of all *S. aureus* isolates. However, each sRNA presented its own distinct expression pattern. A significant correlation, depending on the type of infection or colonization, could not be assigned to each sRNA, except for RsaA and RsaH, where a slight but significant difference was detected depending on the isolation site (Figure 2). These subtle differences in sRNA expression might reflect subtle differences in the genetic content of the various isolates. However, such differences could not be detected using DNA microarrays targeting 180 genes, which showed that the three groups of isolates were genotypically indistinguishable (Figure 1). Notably, the nasal isolates could not be distinguished from the two groups of infection isolates, which is consistent with other studies showing that the nasal carriage and clinical isolates of *S. aureus* belong to the same clades [31].

When considering the sRNA expression kinetics, the most common pattern was an accumulation in the late-exponential growth phase, except for RsaE, which accumulated in the mid-exponential growth phase (Figure 2). RsaE is involved in the regulation of metabolic enzymes involved in the TCA cycle and the folate-dependent one-carbon metabolism [15]. Furthermore, the overexpression of RsaE causes a growth defect that is alleviated by the non-preferred source acetate [19]. Since RsaE is induced in a pre-stationary phase in all clinical strains, an expected role for this sRNA would be to coordinate downregulation of energy metabolism (TCA cycle), of cofactors, vitamins, and purine biosynthesis (folate-dependent one-carbon metabolism) to facilitate adaptation to the entry into stationary phase [15], [19]. The expression of RsaA is under the control of sigmaB, suggesting that the sRNA might be involved in stress responses, biofilm formation, and/or virulence (Figure S2), [15]. The functions of RsaG and RsaH are presently under study. The observed expression profile of the five sRNAs is similar to that observed for strain N315 [19] but slightly different from that for strain RN6390, which carries an *rbsU* mutation (Figure S2), [15]. Indeed, the expression of RsaE was higher in the late-exponential phase in RN6390, while RsaA was poorly expressed. Interestingly, the repair of sigmaB restored the level of expression of RsaA in strain HG001 to a level similar that observed in the clinical isolates (Figure S2), [15]. For RsaE, a similar profile was observed in strains RN6390 and HG001 (Figure S1), revealing that the impairment of the sigmaB regulon in strain RN6390 was not sufficient to explain the altered expression pattern of RsaE. Several other mutations in the RN6390/8325-4 strain genetic background [32] might explain these divergent profiles. When comparing the mean expression levels of the different sRNAs, RNAIII was clearly the most highly expressed RNA in the late-exponential growth phase (Figure 2), an observation that could not be attributed to a better stability of RNAIII since its half-life (45′) is in the range of the four Rsa studied (30–60′) [12], [15]. However, the expression of RNAIII varied up to one thousand times among the various isolates regardless of the calibrator used for comparison (i.e., *gyr*B, 4.5 S and RNase P) (Figure S2). This variability might reflect the previous observations that not all *S. aureus* strains produce equivalent levels of RNAIII, and that *agr*+ and *agr*− heterogeneous populations of isolates co-exist [33]. The levels of RNAIII directly measured in the clinical samples demonstrated that RNAIII is generally much less expressed *in vivo* than in cultures grown to late-exponential phase. This result is observed in all three types of samples, and similar results have been previously reported in various clinical samples [22]–[24]. This result indicates that the cell density in the clinical samples was probably under the threshold for *agr* activation, and corresponds to cultures grown to the mid-exponential phase rather than to cultures grown to the late-exponential phase. However, *agr*-driven quorum sensing does not explain the kinetics observed for the other sRNAs whose expression is independent of *agr* [15].

Overall, the *in vivo* expression levels of RNAIII, RsaA and RsaE resembled those obtained during *in vitro* culture to the mid-exponential phase, while for RsaG and RsaH, the expression resembles *in vitro* culture at the late-exponential phase. The major conclusion drawn from these observations is that *in vitro* culture, whatever the stage of growth, does not actually mimic clinical conditions. These results are consistent with other studies demonstrating that the level of expression of RNAIII in the cystic fibrosis sputum was similar to the low-level *in vitro* expression at the exponential phase, although protein A, which is directly repressed by RNAIII [12], did not reach the expected high level of expression usually observed during the exponential phase [34].

Interestingly, the expression levels of all five sRNAs were extremely variable in abscess samples, while the samples obtained from the CF infection and nasal colonization showed modest and highly uniform sRNA expression, respectively (Figure 3C). This observation might be due to the fact that the bacteria are exposed to more quantitatively variable and diverse stresses during acute infection than during colonization. However, it could also be due to the inherent variability of the external conditions in abscesses (e.g., time after onset of infection), whereas such parameters might be less critical when sampling chronically infected patients or nasal carriers. The striking uniformity of the sRNA expression observed in the nasal samples does not result from the intrinsic properties of the isolates because they display a diversity of expression similar to the other isolates when grown *in vitro* (Figure 3A, 3B). We speculate that the uniform sRNA expression in nasal sample reflects the perfect adaptation of *S. aureus* to the anterior nares in which the bacteria has evolved to minimize the effect of the possible stresses encountered in this niche. Thus the uniform level of sRNA expression in nasal colonization might reflect a sort of vigilance status of the bacteria associated with commensalism. Indeed, Burian *et al.* [24] studied the transcriptional response of several staphylococcal genes from the nose of four persistent carriers and showed that *rec*A (a key enzyme of the SOS response) and *rel*A (a key factor of the stringent response) were expressed at lower levels compared with those found in cells grown in culture medium.In conclusion, we showed the different expression profiles of five sRNAs from 60 different *S. aureus* strains, which were isolated from acute and chronic infections and nasal colonization. The expression profiles were rather strain specific and not correlated to the type of infection from which the bacteria were isolated. The response of each sRNA was not related to a common culture stage and was highly variable. The fact that all five sRNAs were expressed by all of the *S. aureus* strains observed might indicate that these sRNAs regulate targets that are conserved among strains. Studies of their functions are currently underway. The comparison of infection (acute or chronic) *versus* colonization suggests that the sRNA expression levels observed in infection are a consequence of the great variability of the host response, while colonization is apparently a more uniform and *serene* scenario, which might reflect the fact that *S. aureus* is primarily a commensal of the anterior nares.

## Materials and Methods

### Ethics statement

The approval for the collection of clinical samples was obtained in accordance with the ethical chart of the ethic committees (*Comité de protection des personnes Sud-Est IV*) rules and in accordance with French Government regulation (bioethics law 2004-800 of August 6, 2004) which does not require written consent for such study as long as patients did not manifest an opposition to the study and that clinical samples are analyzed anonymously. The study was approved by our institutional board. The collection was declared by the Ministry of Research under the agreement n°DC-2008-176.

### Collection samples and bacterial analysis

Twenty purulent samples were recovered from patients who presented acute suppurative cutaneous infection requiring drainage in the emergency department of Edouard Herriot Hospital (Lyon, France). Upon abscess drainage, a sample of the pus was collected and taken to the bacteriology laboratory for culture. After inoculation of the sample onto a blood agar plate (bioMérieux), Trizol LS reagent (Invitrogen) was added to an aliquot (≈100 to 200 µl) of the sample and the mixture was frozen at – 80°C until extraction. Twenty sputum samples from CF patients infected with *S. aureus* were assayed. The sputum samples were obtained from patients who repeatedly tested positive for *S. aureus* infection for several months, indicating chronic lung infection. These samples were collected during routine visits at the pediatric hospital (Lyon, France), and a routine bacteriological analysis was conducted using established procedures. Subsequently, an aliquot of the sputum sample was treated with Trizol reagent and stored at – 80°C until RNA isolation. The nostrils of 20 healthy *S. aureus* carriers were swabbed using a wool cotton swab moistened with 250 µl of nuclease-free water. The swab was immediately transferred to water and vigorously vortexed. A 10 µl aliquot of the sample was spread onto chromogenic medium selective for *S. aureus* (CHROMagar, Biolys). Trizol reagent (1 ml) was added to the tube containing the swab and vigorously vortexed. The samples were frozen at – 80°C until RNA isolation.

The antimicrobial susceptibility was determined as recommended by the French Society for Microbiology [35].

### Bacterial genomic DNA isolation

Chromosomal DNA were obtained from bacterial cultures grown in Brain Heart BH medium at 37°C for 3 hours. After centrifugation at 3,450× g for 10 min, the bacterial pellet was resuspended in a Tris-HCL buffer (1 mM) containing Triton X-100, lysostaphin (1 mg/ml), lysozyme (10 mg/ml), and ribonuclease A. The mixture was incubated at 37°C for 30 min, and subsequently, proteinase K and buffer AL (DNeasy kit, QIAgen) were added. The DNA was purified using the QIAcube instrument (Qiagen) according to the manufacturer's tissue lysis protocol.

### DNA microarray analysis

The DNA microarray analyses (StaphyType, Alere Technologies, Jena, Germany) were performed according to the protocol described by Monecke *et al.* [36] to characterize the 60 *S. aureus* isolates. The DNA microarrays detected a total of 330 target sequences corresponding to 180 genes and their allelic variants. The data were interpreted based on the algorithm previously described [37]. A split network tree was generated using the modified Parcimony method and the SeaView program version 4 [38].

### RNA isolation from human samples

One milliliter of the samples was transferred to tubes containing Lysing Matrix B (MP Biomedicals). Subsequently, the samples were lysed using a FastPrep Shaking device (40 seconds at a setting of 6.0), followed by the purification and precipitation of the RNAs according to the manufacturer's recommendations (MP Biomedicals). For the nasal swabs, an additional precipitation step was conducted as described [24]. To remove DNA contamination, all of the RNA samples were digested with DNase I (Fermentas) (1 U/1 µg of total RNA at 37°C for 30 min). The absence of DNA contamination was verified by PCR.

### RNA isolation from *S. aureus* isolates

Fresh BH broth was inoculated with an overnight culture to an initial OD_600_ of 0.05 and grown to the mid (OD_600_ = 0.5) or post-exponential phase (OD_600_ = 6) at 37°C with shaking (200 rpm). The bacterial cells were harvested, washed in 10 mM Tris buffer and diluted to a standard OD_600_ of 1.0 (ca 10^9^ cells/ml). One milliliter of diluted bacterial suspension was treated with 20 µl lysostaphin (1 mg/ml). The RNA isolation was performed using the RNeasy Plus mini kit (QIAGEN) according to the manufacturer's instructions.

### Relative quantification of sRNA by RT-PCR

The RNA was quantified using a NanoDrop spectrophotometer, and 1 µg of total RNA was transcribed into cDNA using Enhanced Avian Reverse Transcriptase (Sigma) or Superscript III Reverse Transcriptase (Invitrogen) and the specific primers listed in Table S1. Subsequently, the cDNA was used as a template for the real-time PCR amplification using a LightCycler instrument (Roche) and the specific primers shown in Table S1. The amplification products were detected using SYBR Green. The relative amounts of amplicons for each gene were determined using quantitative PCR relative to an internal standard (*gyr*B encoding GyraseB subunit, 4.5S encoding the ribosomal 4.5S subunit or *rnp*B encoding RibonucleaseP B subunit) [39], [40]. The expression levels of the investigated sRNA were expressed as n-fold differences relative to the calibrator, which were calculated using RealQuant software (Roche Diagnostics). After the normalization of each ncRNA transcripts against the calibrator, the sRNA expression levels *in vivo* were expressed as n-fold differences relative to sRNA levels obtained *in vitro*. The RT-PCR experiments were processed in duplicate for each of the clinical samples and corresponding isolates obtained.

### Statistical analysis

The statistical analyses were performed using SPSS software (version 12.0) and the Mann-Whitney and Levene tests. A value of p<0.05 was considered to be statistically significant.

## Supporting Information

Figure S1 **Kinetics of sRNAs expression** ***in vitro*** **in laboratory strains.** Strains RN6390 (sigmaB defective, plain line) and HG001 (sigmaB restored, dotted line) were grown in BH media until the mid- (OD_550_ = 0.5) (open triangle) or late-exponential (OD_550_ = 6) (plain triangle) growth phases. Total RNA was extracted, and the sRNA transcripts were quantified using RT-PCR normalized to *gyr*B mRNA expression. The results represent the mean of 2 replicate experiments.
(TIF)

Figure S2 **Kinetics of RNAIII expression** ***in vitro*** **according to three different calibrators.** Nine clinical *S. aureus* strains, selected as representatives of the diversity of expression levels observed using the calibrator *gyr*B, were grown in BH media until the mid (OD_550_ = 0.5) (open shape) and late-exponential (OD_550_ = 6) (plain shape) growth phases. Total RNA was extracted, and the RNAIII transcript levels were quantified using RT-PCR normalized to *gyr*B mRNA, 4.5S RNA and *rnp*B sRNA expression.
(TIF)

Table S1 **primers used for reverse transcription and qPCR on LightCycler.**
(DOCX)
