# Supplementary figures and images for: The Expression of Small Regulatory RNAs in Clinical Samples Reflects the Different Life Styles of Staphylococcus aureus in Colonization vs. Infection
Source: PLoS One. 2012 May 22;7(5):e37294. doi: 10.1371/journal.pone.0037294 (PMC3358344; doi:10.1371/journal.pone.0037294)

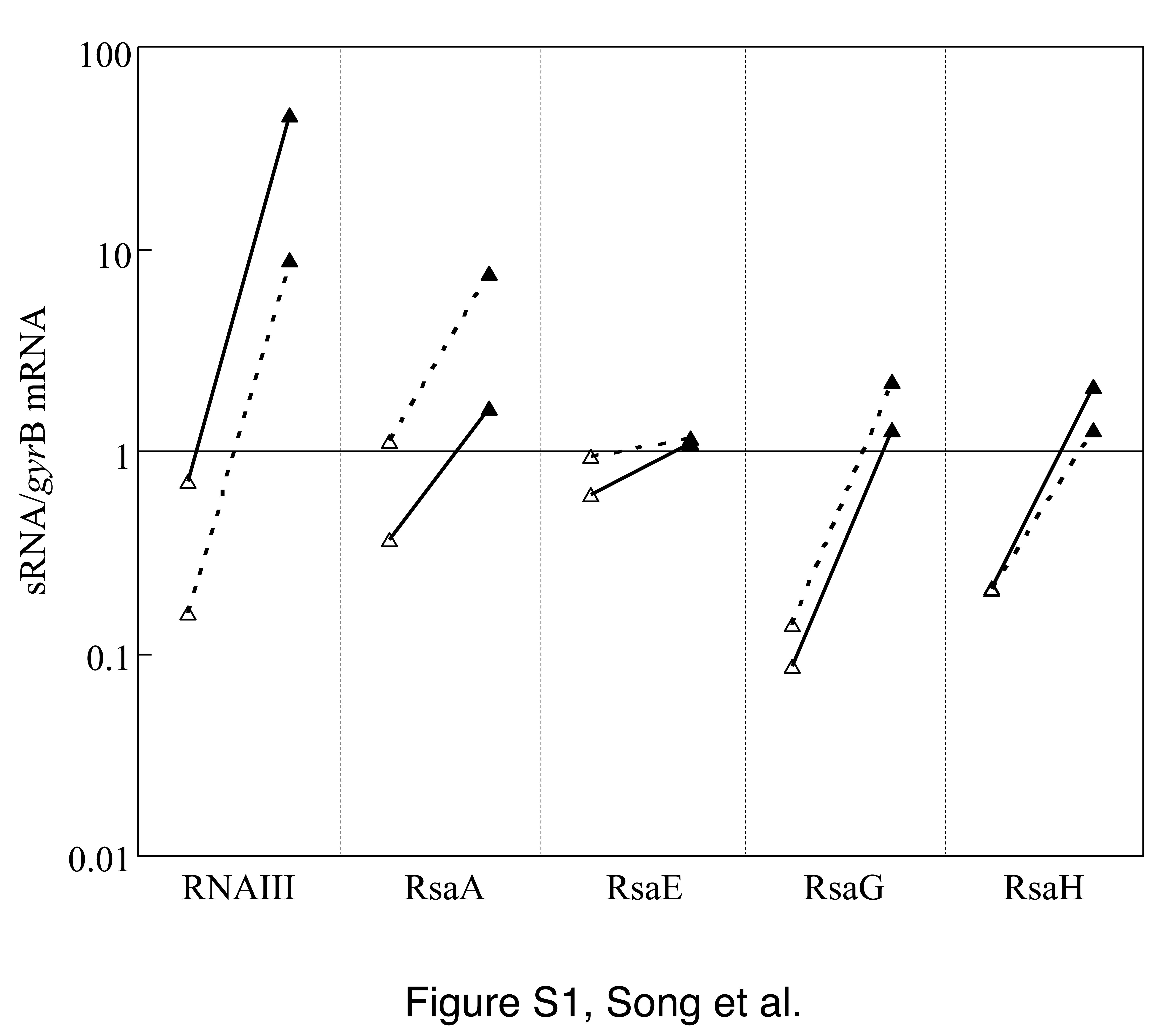

Supplement: Figure S1 — Kinetics of sRNAs expression in vitro in laboratory strains. Strains RN6390 (sigmaB defective, plain line) and HG001 (sigmaB restored, dotted line) were grown in BH media until the mid- (OD550 = 0.5) (open triangle) or late-exponential (OD550 = 6) (plain triangle) growth phases. Total RNA was extracted, and the sRNA transcripts were quantified using RT-PCR normalized to gyrB mRNA expression. The results represent the mean of 2 replicate experiments. (TIF) [file pone.0037294.s001.tif]

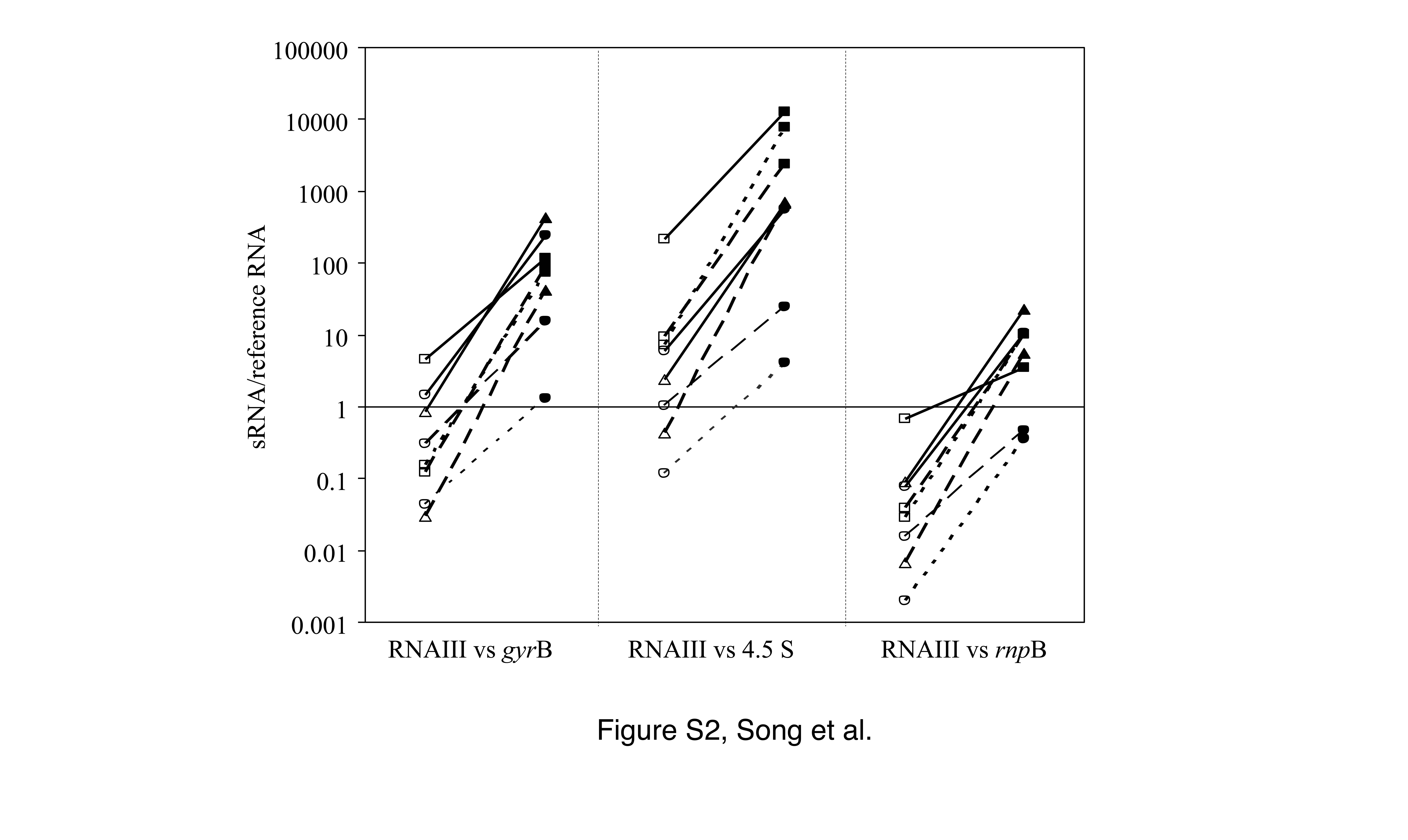

Supplement: Figure S2 — Kinetics of RNAIII expression in vitro according to three different calibrators. Nine clinical S. aureus strains, selected as representatives of the diversity of expression levels observed using the calibrator gyrB, were grown in BH media until the mid (OD550 = 0.5) (open shape) and late-exponential (OD550 = 6) (plain shape) growth phases. Total RNA was extracted, and the RNAIII transcript levels were quantified using RT-PCR normalized to gyrB mRNA, 4.5S RNA and rnpB sRNA expression. (TIF) [file pone.0037294.s002.tif]
