# Supplementary material for: The Expression of Small Regulatory RNAs in Clinical Samples Reflects the Different Life Styles of Staphylococcus aureus in Colonization vs. Infection
Source: PLoS One. 2012 May 22;7(5):e37294. doi: 10.1371/journal.pone.0037294 (PMC3358344; doi:10.1371/journal.pone.0037294)
Supplement: Table S1 — primers used for reverse transcription and qPCR on LightCycler. (DOCX) [file pone.0037294.s003.docx]

**Table S1**: primers used for reverse transcription and qPCR on LightCycler

| Primer | Sequence | References |
| --- | --- | --- |
| GyrB-F  GyrB-R  RNAIII-F  RNAIII-R  RsaA-F  RsaA-R  RsaE-F  RsaE-R  RsaG-F  RsaG-R  RsaH-F  RsaH-R | GGTGGCGACTTTGATCTAGC  TTATACAACGGTGGCTGTGC  GGGATGGCTTAATAACTCATAC  GGAAGGAGTGATTTCAATGG  GTTAACCATTACAAAAATTGTATAGAG  TCTGAATACACGACGCTAAA  AATCACATAACAAACATACCC  TCTGAATACACGACGCTAAA  ATGTGCGTGAAGAGATGAAAGA  CTAAGTCGGGCAAATAAGGATAC  TAAAACGCTGCATGATACAAAC  CCACTACTAAAGGGAGTCAAA | [29]  [29]  [30]  [30]  This work  This work  This work  This work  This work  This work  This work  This work |
